# Overexpression of the Orotate Phosphoribosyl-Transferase Gene Enhances the Effect of 5-Fluorouracil in Head and Neck Squamous Cell Carcinoma *In Vitro*

**DOI:** 10.1155/2012/649605

**Published:** 2012-03-26

**Authors:** Ryuji Yasumatsu, Torahiko Nakashima, Shizuo Komune

**Affiliations:** Department of Otorhinolaryngology, Graduate School of Medical Sciences, Kyushu University, 3-1-1 Maidashi, Higashi-ku, Fukuoka 812-8582, Japan

## Abstract

5-Fluorouracil (5-FU) is a widely used drug in head and neck squamous cell carcinoma (HNSCC). In the anabolic pathway of 5-FU, the first step in activation of the drug is phosphorylation of 5-FU by orotate phosphoribosyltransferase (OPRT), which directly metabolizes 5-FU to 5-fluorouridine monophosphate (FUMP) in the presence of 5-phosphoribosyl-1-pyrophosphate. To date, OPRT expression in the tumors has been related to the clinical response or survival of cancer patients receiving 5-FU-based chemotherapy. In this study, we examined whether OPRT expression correlates with the chemosensitivity to 5-FU and cell proliferation in HNSCC. We constitutively expressed an OPRT cDNA in an HNSCC cell line. The effects of OPRT expression on *in vitro* cell growth and 5-FU cytotoxicity were examined. OPRT transfection increases the cytotoxicity of 5-FU without affecting cell proliferation of HNSCC cells *in vitro*. These results indicate that OPRT expression plays an important role in the sensitivity of HNSCC to 5-FU chemotherapy.

## 1. Introduction

5-Fluorouracil (5-FU) has been used most frequently for treating head and neck squamous cell carcinoma (HNSCC) in a form of single agent or in combination with cisplatin [1] and the drug of choice for systemic therapy in colorectal cancer [2]. However, nowadays 5-FU resistance during the course of treatment has become common, which is an important cause of failure for cancer therapies [3].

It has been reported that response rate of 5-FU and its derivatives are due to interindividual difference in the enzyme activities for anabolism and catabolism. In the anabolic pathway of 5-FU, the first step in activation of the drug is phosphorylation of 5-FU by orotate phosphoribosyltransferase (OPRT), which directly metabolizes 5-FU to 5-fluorouridine monophosphate (FUMP) in the presence of 5-phosphoribosyl-1-pyrophosphate [4]. This step is the most important mechanism of 5-FU activation. To date, OPRT expression in the tumors has been related to the clinical response or survival of cancer patients receiving 5-FU-based chemotherapy [5, 6]. However, no study has confirmed directly whether the regulation of intratumoral OPRT expression level affects the efficacy of 5-FU and the cell activity in HNSCC. We therefore investigated whether overexpression of the OPRT enhances sensitivity to 5-FU.

In this study, to assess the role of OPRT in the biological regulation of HNSCC, we constitutively expressed the OPRT complementary DNA (cDNA) in HNSCC cell line. The effect of OPRT on *in vitro* cell growth and 5-FU cytotoxicity was examined.

## 2. Materials and Methods

### 2.1. Cell Line

 The human head and neck squamous cell carcinoma cell line, YCU-H, which was generously provided by Dr. M. Tsukuda, was cultured in RPMI 1640 medium and supplemented with 10% fetal bovine serum (FBS) and penicillin/streptomycin 1000 IU/mL (Invitrogen, Carlsbad, CA, USA). Cells were maintained in a humidified incubator at 37°C under 5% CO_2_.

### 2.2. Vector Construction and Transfection

Full-length human OPRT cDNA (kindly provided by Taiho Pharmaceutical Co. Ltd., Tokyo, Japan) that contained the entire coding sequence was subcloned in its sense orientation into Eco RI-Kpn I sites of the expression vector pTARGET. Human YCU-H cells were stably transfected with the pTARGET-OPRT plasmid and the pTARGET vector control plasmid via liposome-mediated transfection using Lipofectamine 2000 (Invitrogen), according to the conditions described by the supplier. Forty eight hours after transfection, transduced cells were selected in complete medium containing 500 *μ*g/mL Geneticin (Invitrogen) for 2 to 3 weeks. After selection, single independent clones were randomly isolated using cloning rings, and each clone was plated separately.

### 2.3. Western Blot Analysis of Cultured Cells

Western blot analyses were performed as reported previously [7, 8] to detect the OPRT expression in the YCU-H cell line. Total cellular protein was extracted and quantified using the M-Per Mammalian Protein Extracted Reagent and “Coomassie” Protein Assay Reagent Kit (Pierce, Rockford, IL). Equal amounts (10 *μ*g) of cell lysates were subjected to SDS-polyacrylamide gel electrophoresis and transferred to a nitrocellulose membrane. The membrane was blocked for 1 h with 5% nonfat dry milk in PBS and then incubated with the purified polyclonal antibody against OPRT (kindly provided by Taiho Pharmaceutical Co. Ltd., Tokyo, Japan) or an anti-*β*-actin antibody (Sigma Chemical Company, St. Louis, MO, USA) for loading control for 1 h at 37°C. The membranes were then incubated with the HRP-conjugated goat anti-rabbit Ig G secondary antibody for 1 h at room temperature, followed by the detection with the enhanced chemiluminescence (ECL) system (Amersham International, Buckinghamshire, UK).

### 2.4. *In Vitro* Proliferation Assays

Transfected clone and vector control clone cells (1 × 10^4^ cells/dish) were seeded onto 35 mm dishes in RPMI 1640 medium plus 10% FBS. The number of the cells was counted every 48 hours for 8 subsequent days, in triplicate assays, using a Coulter Counter (Beckman Coulter, Fullerton, CA, USA). The mean values were used to generate growth curves.

### 2.5. Drug Sensitivity Assay

Transfected cells and control cells were plated in 96-well plates at a density of 10^4^ cells/well and further incubated for 24 h. The medium was then removed and replaced with fresh medium containing 5-FU (kindly provided by Kyowa Hakko Co. Ltd.) for another 48 h. Then, 10 *μ*L sterile MTT dye (3-[4,5-dimethylthiazol-2-yl]-2,5-diphenyltetrazolium bromide, 5 mg/mL; Sigma) was added to the culture medium to a final concentration of 0.5 mg/mL and incubated at 37°C for 2 h. After that, the formazan crystals were solubilized with 100 *μ*L of dimethylsulfoxide (DMSO) for 10 min. Spectrometric absorbance at 550 nm was measured with microplate reader. The IC_50_ value was determined by the dose of drug that caused 50% cell viability.

### 2.6. Statistical Analysis

Statistical analyses were performed using the Mann-Whitney *U* test. The Kaplan-Meier method was used for analysis of survival data. The significance of differences of survival plots was analyzed by the log-rank test. Differences with a *P* value <0.05 were considered to be significant.

## 3. Results

### 3.1. Transfection of OPRT cDNA in YCU-H Human Head and Neck SCC Cell Line

**Figure 1:**
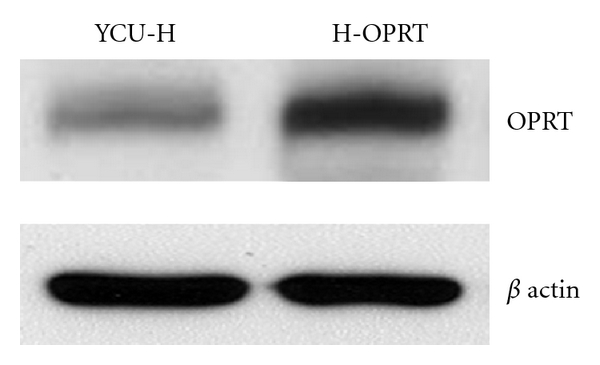
Western blot analysis indicating OPRT expression levels in human head and neck squamous cell carcinoma YCU-H cells transfected with OPRT cDNA. The OPRT protein level was increased 35 times in the OPRT transfected clones H-OPRT in comparison with the parental cell line, YCU-H cells.

Four independent clones were selected after 2 to 3 weeks of growth in medium supplemented with Geneticin (500 *μ*g/mL). Representative clone, H-OPRT, was selected for use in subsequent experiments. The levels of OPRT of the selected clone and YCU-H cells are illustrated in Figure 1. The level of OPRT protein was increased 35 times in the H-OPRT cells compared to control cells, respectively.

### 3.2. *In Vitro* Growth of the OPRT Overexpressing Cell Line

**Figure 2:**
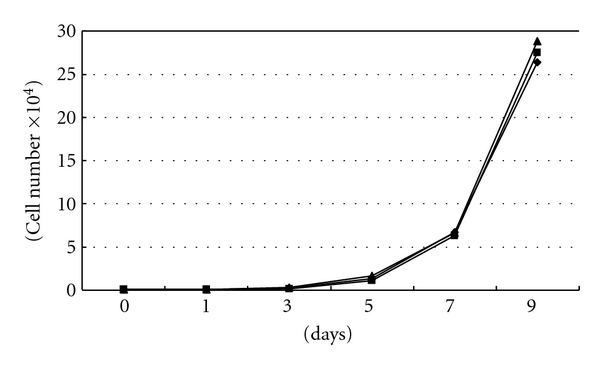
*In vitro* growth curve of the OPRT transfectants. There were no significant differences in *in vitro* growth between OPRT clone and control clones. ◆: YCU-H, ■: pTARGET, ▲: H-OPRT.

 When grown in 10% fetal bovine serum medium, the vector control cell lines and OPRT transfected cell line showed similar doubling times. There were no significant differences in *in vitro* growth between OPRT overexpressing clone and control clones (Figure 2). 

### 3.3. Correlation between the Level of OPRT Expression in HNSCC Cells and Their Sensitivity to 5-FU

**Figure 3:**
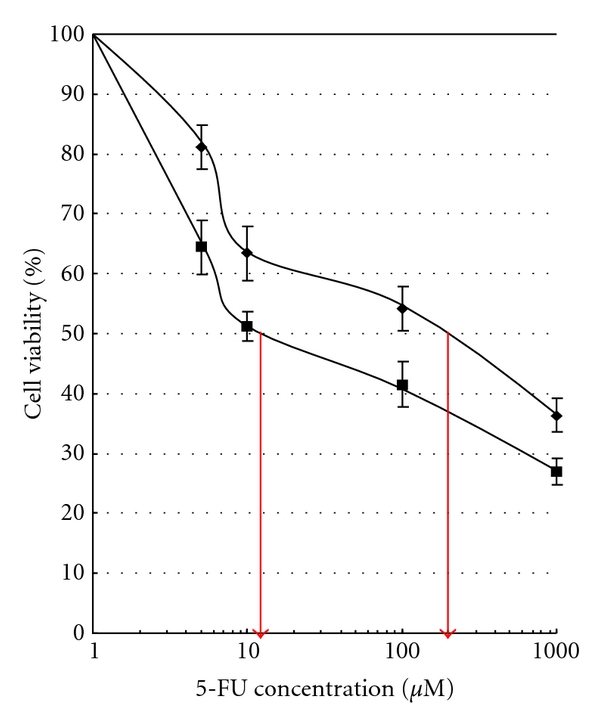
The cytotoxic effect of 5-FU was measured by MTT assay. IC_50_ values were estimated from the regression line of log-logit plots of concentration versus growth inhibition rate. Twenty-four hours after plating, the cells were exposed to 5-FU for 2 days. Each value represents the mean of triplicate plates; bars, SD. ◆: pTARGET, ■: H-OPRT.

Cell cytotoxic assays were performed using MTT assay to examine whether the transfected OPRT cDNA increased 5-FU sensitivity in the OPRT overexpressing cells. The increased sensitivity of the OPRT transfected cells to 5-FU was observed in H-OPRT cells. The 50% growth inhibitory (IC_50_) value to 5-FU in H-OPRT cells was 11 *μ*M, which was lower than those of control cells (IC_50_: 150 *μ*M) (Figure 3).

## 4. Discussion

For decades, 5-FU and its derivatives such as 5′-DFUR and tegafur have been used to treat cancer patients, and the effectiveness of 5-FU is well proven in HNSCC. However, the presence of drug-resistant tumor cells, which occurs with other chemotherapeutic agents as well, causes poor response to 5-FU-based chemotherapy [3].

To phosphorylate 5-FU into its nucleotides, the following 3 metabolic pathways have been reported: pathway 1: phosphorylation to 5-fluorouridine monophosphate (FUMP) by OPRT; pathway 2: phosphorylation to 5-fluorodeoxyuridine (FdUR) by TP and a sequent conversion to 5-fluorodeoxyuridine-monophosphate (FdUMP) by thymidine kinase (TK); pathway 3: phosphorylation to 5-fluorouridine (FUR) by UP and a sequent conversion to FUMP by uridine kinase (UK) [9, 10]. Thus, OPRT is one of the main enzymes responsible for the phosphorylation of 5-FU in human cancer cells. In addition, several clinical reports have demonstrated a relationship between OPRT activity in the tumors and clinical response of cancer patients receiving 5-FU-based chemotherapy [5, 6]. However, the role of OPRT expression in HNSCC has not been established and, to the best of our knowledge, there are no studies investigating the relationship between the *in vitro* OPRT expression and 5-FU cytotoxicity in HNSCC.

To directly confirm whether the overexpression of OPRT affects the sensitivity to 5-FU and to review the role of OPRT in HNSCC cells, we transfected OPRT cDNA in a human HNSCC cell line, YCU-H. As a result, we found a 35-fold higher expression of the endogeneous OPRT protein in OPRT transfected YCU-H cells. The present study also demonstrated that the increased sensitivity to 5-FU was observed in OPRT overexpressing cells. Inaba et al. reported a significantly low activity of OPRT in the 5-FU-resistant cell line by measuring the enzyme activities in the human cancer cell lines, [11]. Taomoto et al. also indicated that OPRT overexpression plays an important role in the increased sensitivity of gastric carcinomas to 5-FU chemotherapy [4]. The present result is compatible with those of the earlier studies. These results, combined with other reports, strongly suggest that overexpression of the OPRT in cancer cells enhances the sensitivity to 5-FU treatment.

There were no significant differences in the *in vitro *growth between OPRT overexpressing cells and control cells in this study. These results show that overexpression of OPRT protein is not linked to rapid tumor cell proliferation of HNSCC cells. On the other hand, in bladder carcinoma, it was reported that OPRT activity was upregulated compared with the activity in normal bladder and that OPRT may be of prognostic value [12]. Miyake et al. also reported that OPRT may play a potential role in regulating the malignant potential of pancreatic cancer [13]. Because the regulation of OPRT expression and its involvement with tumor proliferation remains unclear, we could not reach any conclusion about the relationship between this enzyme and tumor progression.

In conclusion, our data suggest that OPRT affects the chemotherapeutic effect of 5-FU in HNSCC cells *in vitro*. The present results strongly indicate that OPRT overexpression plays an important role in the sensitivity of HNSCC to 5-FU chemotherapy. Because the level of OPRT expression could be used as a predictive indicator for 5-FU efficacy against HNSCC, the accurate prediction of 5-FU efficacy may help to select patients for more intensive treatment including CDDP based chemoradiotherapy.
